# Human amniotic mesenchymal stem cells improve the follicular microenvironment to recover ovarian function in premature ovarian failure mice

**DOI:** 10.1186/s13287-019-1315-9

**Published:** 2019-10-02

**Authors:** Rongxia Liu, Xiaoyu Zhang, Zhenhai Fan, Yuying Wang, Guanping Yao, Xue Wan, Zulin Liu, Bing Yang, Limei Yu

**Affiliations:** 1 Key Laboratory of Cell Engineering in Guizhou Province, The Affiliated Hospital of Zunyi Medical University, Zunyi City, 563003 China; 2 Biological Treatment Talent Base of Guizhou Province, The Affiliated Hospital of Zunyi Medical University, Zunyi City, 563003 China; 3 Zunyi Stem Cell and Regenerative Medicine Engineering Research Center, The Affiliated Hospital of Zunyi Medical University, Zunyi City, 563003 China; 4 Reproductive Center, The Affiliated Hospital of Zunyi Medical University, Zunyi City, 563003 China; 5 Department of Gynecology, The Affiliated Hospital of Zunyi Medical University, Zunyi City, 563003 China

**Keywords:** Premature ovarian failure, Hydrogen peroxide, Human amniotic mesenchymal stem cells, Diethylstilbestrol, Reproduction, Ovary, Microenvironment

## Abstract

**Background:**

Many adult women younger than 40 years old have premature ovarian failure (POF) and infertility. Previous studies confirmed that different tissue-derived stem cells could restore ovarian function and folliculogenesis in chemotherapy-induced POF mice. The aim of this study was to explore the therapeutic efficacy and underlying mechanisms of human amniotic mesenchymal stem cells (hAMSCs) transplantation for hydrogen peroxide-induced ovarian damage.

**Methods:**

Bilateral ovaries of female mice were burned with 10% hydrogen peroxide to establish a POF model. After 24 h of treatment, hAMSCs and diethylstilbestrol were administered to POF mice by intraperitoneal injection and intragastric administration, respectively. After either 7 or 14 days, ovarian function was evaluated by the oestrus cycle, hormone levels, ovarian index, fertility rate, and ovarian morphology. The karyotype was identified in offspring by the G-banding technique. hAMSCs tracking, immunohistochemical staining, and real-time polymerase chain reaction (PCR) were used to assess the molecular mechanisms of injury and repair.

**Results:**

The oestrus cycle was recovered after hAMSCs transplantation at 7 and 14 days. Oestrogen levels increased, while follicle-stimulating hormone levels decreased. The ovarian index, fertility rate, and population of follicles at different stages were significantly increased. The newborn mice had no obvious deformity and showed normal growth and development. The normal offspring mice were also fertile. The tracking of hAMSCs revealed that they colonized in the ovarian stroma. Immunohistochemical and PCR analyses indicated that changes in proteins and genes might affect mature follicle formation.

**Conclusions:**

These results suggested that hAMSCs transplantation can improve injured ovarian tissue structure and function in oxidatively damaged POF mice. Furthermore, the mechanisms of hAMSCs are related to promoting follicular development, granulosa cell proliferation, and secretion function by improving the local microenvironment of the ovary.

## Background

Premature ovarian failure (POF) is a gynaecological endocrine disease characterized by abnormal oestrogen levels and gonadotropin, which manifests as irregular menstruation, amenorrhea, infertility, and perimenopause syndrome affecting women younger than 40 years of age. Approximately 1% of women under the age of 40 years could develop POF [1]. The reasons for POF may be varied, including genetic predisposition, autoimmune conditions, infections, and iatrogenic causes [2]. Long-term health consequences, including psychological distress, infertility, osteoporosis, heart disease, autoimmune disorders, and increased mortality, have significant impacts on the quality of life for women diagnosed with POF [3]. The mechanisms of POF genesis and progression involve follicle atresia, granulosa cell apoptosis, interstitial fibrosis, and disturbed sex hormone levels. There may also be an imbalance in immune function and inflammatory responses [4, 5]. Therefore, in addition to increased follicle-stimulating hormone (FSH) and decreased oestrogen and anti-Müllerian hormone(AMH) levels in the blood circulation, changes in the expression of a series of molecules also occur in the local ovary [6, 7], including a variety of follicular development-related growth factors, such as the forkhead box L2 gene (FOXL2), octamer combination transcription factors (Oct4), growth differentiation factor-9 (GDF-9), leukaemia inhibitory factor (LIF), and stem cell factor (SCF). Thus far, there are limitations of hormone replacement therapy, in vitro oocyte maturation or oocyte/ovarian cryopreservation for transplantation on POF [1, 3]. However, no radical cure is yet available for reversing the POF to a normal ovarian structure and function. There is an urgent need to improve the current treatment strategies.

Stem cell therapy has been suggested as a promising measure in the treatment of several human diseases and applications of regenerative medicine because of the self-renewal and differentiation abilities of these cells, which can replace the damaged tissue, or the paracrine cytokines and exosomes, which can rescue injured tissues [8, 9]. Recent studies have shown that bone marrow mesenchymal stem cells (MSCs) [10–14], amniotic fluid stem cells [15–18], adipose-derived stem cells [19, 20], human umbilical cord MSCs [21, 22], and menstrual blood stem cells [23–25] can restore ovarian function and fertility in mice models of POF. However, many of the suitable cell types currently identified for clinical application involve invasive procedures or have a low magnitude of the original stem cells.

Amnion is a waste product of perinatal tissue sources so the procedure to obtain human amniotic mesenchymal stem cells (hAMSCs) is noninvasive and not under ethical debate. hAMSCs may prevent age-related reductions in proliferative and differentiation potential characteristics [26]. hAMSCs have the common characteristics of multipotent MSCs, including self-renewal, high rates of proliferation, multi-differentiation capacity, immunosuppressive and anti-inflammatory effects, and paracrine activity [27–32]. Researchers have reported that hAMSCs secrete significant amounts of various factors, including HGF, IGF-1, VEGF, EGF, GDF-9, bFGF, and many miRNAs [33]. Hence, hAMSCs are an ideal cell type for the treatment of tissue damage. The efficacy of hAMSCs has been demonstrated in several trials, including trials evaluating hAMSCs as a treatment for heart failure, myocardial infarction, Alzheimer’s disease, and spinal cord injury [34–39]. A few studies have reported that hAMSCs can rescue ovarian function in chemotherapy-induced POF mice [40, 41]. However, whether hAMSCs transplantation can restore ovarian function in hydrogen peroxide (H_2_O_2_)-induced POF with oxidative damage is still unknown. In this study, hAMSCs were injected intraperitoneally into H_2_O_2_-induced POF mice to evaluate the restorative effect on ovarian function. The mechanism underlying hAMSCs-mediated repairs was further studied.

## Methods

### Isolation and culture of hAMSCs

Human placentas were obtained from term pregnancies during uncomplicated caesarean sections after written and informed consent was obtained from each woman who tested negative for HIV-I and hepatitis virus B and C. The institutional ethics committee approved the use of human amnions in this study. According to the reported protocol [42], the washed amniotic membrane that was mechanically separated from the chorion portion of the placenta was cut into pieces of 0.5 to 1.0 cm^2^. The amniotic membrane segments were digested twice with 0.05% trypsin with 0.02% EDTA at 37 °C for 40 min with gentle agitation to remove amniotic epithelial cells. The rest of the tissue pieces were placed in LG-DMEM containing collagenase II (0.75 mg/ml; Gibco, USA) and DNase I (0.075 mg/ml; Worthington, USA) and were incubated at 37 °C for 60 min to isolate hAMSCs. The cell suspensions were filtered and centrifuged. The cellular pellets were cultured with LG-DMEM medium supplemented with 10% foetal bovine serum (FBS), l-glutamine (GE Healthcare Bio-Sciences AB, USA), and human FGF basic (10 ng/100 ml) (R&D Systems, USA) and incubated at 37 °C and 5% CO_2_. When cells reached 80–90% confluence, adherent cells were trypsinized and passaged. Third-passage to fifth-passage hAMSCs were used for all of the transplantation experiments.

### hAMSC phenotypic characterization

The hAMSCs-specific surface antigens were stained with FITC-conjugated anti-human CD90, PerCP-Cv5.5-conjugated anti-human CD105, APC-conjugated anti-human CD73, and PE-conjugated anti-human CD44, CD45, CD34, CD11b, CD19, and HLA-DR antibodies (BD Biosciences, USA) or their corresponding isotype control. Then, the stained cells were tested using FACSCalibur flow cytometry (BD Biosciences, USA). A minimum of 2 × 10^4^ cells were used for each experiment, and Cell Quest software was used for data analysis.

### PKH26-labelled hAMSCs

When cells reached 80–90% confluence, adherent hAMSCs were trypsinized and centrifuged to obtain cellular pellets and then resuspended in 1 ml PBS. First, 2.0 × 10^6^ cells were incubated with 250 μl 4.0 × 10^−6^ mol/L PKH26 at room temperature for 5 min. The reaction was stopped with 250 μl FBS and diluted with an equal amount of 10% FBS. The cells were centrifuged and resuspended in 10 mL complete medium and then washed twice with PBS. The morphology of PKH26-labelled hAMSCs was observed under an inverted phase-contrast fluorescence microscope, and the percentage of PKH26 marker cells was detected by flow cytometry.

### Animals

The approval was obtained from the Animal Ethics Committee of Zunyi Medical University. The specific pathogen-free grade Balb/c mice were handled in accordance with the Institute Animal Care and Use Committee at Zunyi Medical University. Eight-week-old male and female Balb/c mice were purchased from the Daping Hospital Animal Experiment Center of the Third Military Medical University (Chongqing, China). Mice were housed in animal facilities and were fed a standard pellet diet with free access to water for 1 week before the experiment.

### POF model establishment

To establish the POF model in mice, 88 female mice were randomly divided into two groups: a normal group (*n* = 22) without any treatment for the normal and POF groups (*n* = 66). Mice were anaesthetized with 10% chloral hydrate (0.35 ml/100 g) via intraperitoneal injection. The abdomen was opened under aseptic conditions, and the bilateral ovaries were burned for 30 s~1 min with 10% hydrogen peroxide and then washed with normal saline. The abdominal wall was sutured. The oestrus cycles were routinely assessed by vaginal smears, and venous blood samples were collected in dioestrus in the normal group and the POF group. Disordered oestrus cycles, low serum oestrogen level, high FSH, and decreased follicle numbers indicated that the POF model was successfully established in mice. The POF mice were randomly divided into 3 groups: the model, diethylstilbestrol (DES), and hAMSCs (hAMSC) treatment groups.

### hAMSCs transplantation

The third- to fifth-passage hAMSCs were collected and stored at room temperature in PBS for less than 30 min before transplantation. In the stem cell-transplanted mice, 1 × 10 ^6^ cells in 300 μl PBS were slowly injected intraperitoneally in POF mice after modelling for 24 h. For comparison, the same volume of isotonic PBS was injected into the POF model and the normal mice. The mice were treated with DES (No. H34021221, Hefei Jiulian Pharmaceutical, Anhui, China) at 0.15 mg/kg/day intragastrically for 14 days in the DES group [43].

### Examination of the oestrus cycle

To evaluate the effect of hAMSCs treatment, vaginal exfoliated epithelial cells were collected using a sterile cotton swab and transferred to a glass slide, which was air-dried, fixed in methanol, and examined after haematoxylin and eosin (H&E) staining. The oestrus cycles of female mice were evaluated by vaginal smears every day for the 14 consecutive days after hAMSC transplantation. The morphological changes of vaginal epithelial cells are shown in Fig. 2. Furthermore, the four phases of the oestrus cycle were determined according to the proportions of leukocytes, nucleated epithelial cells, and cornified squamous epithelial cells by examining vaginal cytology.

### Hormone assay

Under anaesthesia, blood was taken from the eye posterior orbital venous plexus of the mice in the dioestrus stage of the oestrus cycle after hAMSCs transplantation on the 7th and 14th day. Samples were stored at 4 °C for 1 h, and serum was collected following centrifugation at 3000 rpm for 10 min and frozen at − 80 °C before hormone analysis. The levels of oestrogen and FSH were measured according to the manufacturer’s guide using ELISA kits (Elabscience, Wuhan, China). Finally, optical density values of the sample were measured at 450 nm by an IMark Microplate Absorbance Reader (Bio-Rad, USA).

### Ovarian index and fertility estimation

The bilateral ovary and body weights of mice were recorded at 7 and 14 days after hAMSC treatment. The ovarian index was calculated as ovary weight/body weight. Fourteen days after transplantation, 10 female mice in each group were matched with 10 male mice. The presence of a copulatory plug was defined as successful mating. Males were randomly rotated among cages after each pregnancy. The number of female mice that gave birth and the number of offspring per litter were recorded. The fertility rate was calculated based on the number of cases/mated cases.

### Chromosome karyotype analysis

Chromosome karyotype analysis of offspring mice was performed in the bone marrow mononuclear cells. The specific method is as follows. The mice were injected intraperitoneally with 0.04% colcemid (Sigma Aldrich, USA) at 0.1 ml/10 g. After 4 h of treatment, the mice were killed to harvest the femur. The marrow cavity was rinsed repeatedly with PBS to collect bone marrow cells. The samples were filtered through a 300-μm mesh filter to remove debris. Then, bone marrow cells were centrifuged at 1000 rpm for 10 min to obtain cellular pellets. The centrifuged cellular pellets were resuspended in 1.0 mL of chilled methanol-acetic acid fixative after low permeability treatment with 75 mmol/L potassium chloride. The cell suspension was dropped onto a pre-cooled slide and immediately used for chromosome preparation by trypsin-G-banding technology. The karyotype analysis of mouse bone marrow cells was carried out using the CV Cytovision karyotype analysis system (AI, UNK) and Cytovision 3.93 software (Leica Biosystems, Germany). Thirty metaphase mitotic phases were counted, and 5–10 chromosome karyotypes were analysed.

### Histopathological examination and ovarian follicle counting

Ovarian tissue was collected, and the appearance of the ovary was observed with the naked eye. After the removed ovaries were fixed in 4% paraformaldehyde for at least 48 h, the ovaries were dehydrated, paraffin-embedded, serially sectioned at 5 μm depth, and mounted on microscope slides. The sections were stained by H&E, and then, the histopathology of ovarian tissue was observed by using BX51 microscopy (Olympus, Japan). The follicles were counted only on those containing an oocyte with a clearly visible nucleus. The follicles were categorized as primordial, primary, secondary, and atretic follicles according to the method described previously [44].

### PKH26-labelled hAMSCs tracking

To determine the location of the transplanted hAMSCs and their fate in ovarian tissues, immunofluorescence was performed. Three mice in each group were randomly selected to kill at 7 and 14 days after PKH26-labelled hAMSCs transplantation. A side ovary was collected and immersed in 20% sucrose solution for 2 h. The ovary was embedded by frozen section embedding agent. The ovaries were serially frozen sectioned at a depth of 5 μm. All sections were visualized using DMIRB fluorescence microscopy (Leica Biosystems, Germany). The location and distribution of the PKH26-marked hAMSCs were determined by detecting red signals in ovarian tissue.

### Immunohistochemistry

The fixed ovaries with 4% paraformaldehyde were embedded in paraffin. Sections were fixed for 5 min in neutral buffered formalin, after which endogenous peroxidase activity was quenched by incubating in 3% hydrogen peroxide with methanol for 10 min. The antibody retrieval of each section was treated by microwave two times and washing three times with PBS. Different sections were incubated with the following primary antibodies: rabbit anti-mouse vascular endothelial growth factor (VEGF, 1:100, Bo Orson, Beijing, China), rabbit anti-mouse FSH receptor (FSHR, 1:100, ZSGB-BIO, Beijing, China), rabbit anti-mouse insulin-like growth factor-1 (IGF-1, 1:30, Bo Orson, Beijing, China), anti-tumour necrosis factor α (TNF-α, 1:100, ZSGB-BIO, Beijing, China), and anti-interleukin-1β (IL-1β, 1:30, Bo Orson, Beijing, China) at 4 °C overnight. The washed sections were incubated with a goat anti-mouse IgG secondary antibody (1:100; Santa Cruz, CA, USA) at 37 °C for 30 min. The peroxidase substrate was developed by a diaminobenzidine chromogenic kit. Slides were counter-stained with haematoxylin for 60 s. The dehydrated and enclosed sections were photographed with the Leica QwinV3 image analysis system (Leica Biosystems, Germany).

### RT-PCR

Total RNA was extracted from ovary tissues using RNAiso™ Plus (TaKaRa, Dalian, China) according to the manufacturer’s protocol. The complementary DNA was synthesized from qualitative and quantitative RNA with Prime Script RT Reagent Kit (TaKaRa, Dalian, China). The expression levels of FOXL2, OCT4, GDF-9, SCF, and LIF mRNA were assessed by RT real-time PCR with SYBRR Premix Ex Tap™ (TaKaRa, China) and the ABI 7500 real-time PCR system. The reaction conditions for gene amplification were 95 °C for 10 min, 95 °C for 15 s, and then 60 °C for 1 min. All procedures were performed following the manufacturer’s instructions. The specific PCR primers were designed according to DNA sequences in the NCBI database (see Table 1). The level of gene expression was normalized to the reference gene GAPDH. For expression analysis, data from three replicates were analysed by using the 2^-ΔΔCt^ method [45].

**Table 1:** Primer pairs of target genes in real time PCR

Gene	GenBank Acc.	Forward primer	Reverse primer
GAPDH	NM050371	TGTGTCCGTCGTGGATCTGA	TTGCTGTTGAAGTCGCAGGAG
LIF	MA115566	TTGATCCCGACTCAAGCAACC	CTGAAGCCGCTACCATGCAA
GDF-9	MA025612	GTTCCCAAACCCAGCAGAAGTC	GTCCAGGTTAAACAGCAGGTCCA
FOXL2	MA108348	CACCTCCAGGCCAGGTCTTTA	TTTAGCAAACTCCAAGGCCATTAC
OCT4	MA096236	CAGACCACCATCTGTCGCTTC	AGACTCCACCTCACACGGTTCTC
SCF	MA114197	AGATCTGCGGGAATCCTGTGA	CATCCCGGCGACATAGTTGA

### Statistical analyses

The statistical analyses were performed by using SPSS 18.0 software. The results are shown as the mean ± standard deviation (SD) or percentage. One-way ANOVA with LSD tests was used to determine significant intergroup differences. The chi-square test was performed for the percentage*. P* < 0.05 was considered to be significant.

## Results

### Characterization of hAMSCs

**Fig. 1:**
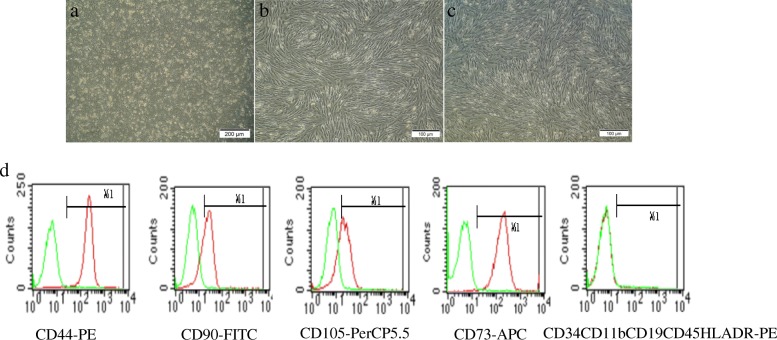
Morphology and molecular phenotype of hAMSCs. The cells morphology of passage 0 (**a**, × 40), 3 and 4 (**b**, **c**, × 100) hAMSCs. **d** Phenotypic analysis of the fourth-passage hAMSCs by flow cytometry

After 48 h in culture, hAMSCs appeared to be spindle-, polygon-, or star-shaped in morphology and reached 50% fusion (Fig. 1a). After subculture for 3 days, the long spindle cells increased significantly. The morphology of passages 3 to 4 hAMSCs was more homogeneous and in a single layer vortex arrangement (Fig. 1b, c). Flow cytometry analysis was used to identify surface marker expression of the fourth-passage hAMSCs. Over 95% of hAMSCs expressed CD44, CD90, CD105, and CD73, and less than 2% of the cells were positive for CD45, CD34, CD11b, CD19, and HLA-DR (Fig. 1d).

### hAMSCs recovered the oestrus cycle

**Fig. 2:**
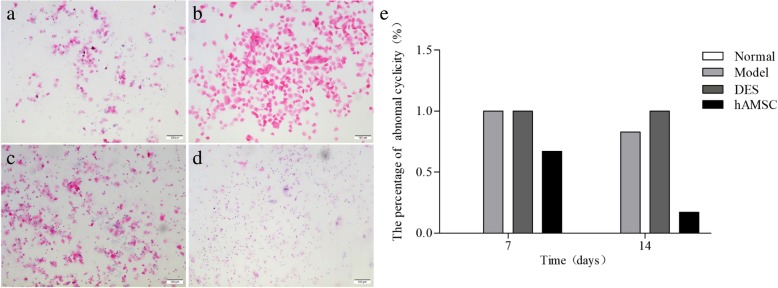
The transplanted hAMSCs improve oestrus cycles in POF mice. Vaginal smears were obtained, and the oestrus cycles were evaluated by H&E staining. Representative photographs for proestrus (**a**), oestrus (**b**), metoestrus (**c**), and dioestrus (**d**) are shown (× 100). **e** The percentage of abnormal cyclicity was detected in mice at 7 and 14 days after cells transplantation (*n* = 6)

Normal female mice have regular oestrus cycles with durations of 4 to 6 days, including proestrus for 1 day, oestrus for 1 to 2 days, metoestrus for 1 day, and dioestrus for 1 to 2 days (Fig. 2a–d). Vaginal exfoliation cells were assessed after H&E staining. Normal oestrus cycles were maintained in the normal group without an abnormal oestrus cycle. In the hAMSC group, 66.67% of the mice still showed an abnormal oestrus cycle at 7 days after hAMSCs transplantation. After transplantation of hAMSCs for 14 days, 83.33% of the mice showed an irregular cycle in the model group. In comparison, abnormal oestrus cycles were only maintained in 16.67% of mice in the hAMSC group (Fig. 2e). These results showed that the irregularity of oestrus cycles was significantly decreased by hAMSCs. However, the numbers of mice with normal cycles in the hAMSC group were significantly lower compared to the normal group. In the DES group, mice were always in the oestrus stage of the oestrus cycle at 7 and 14 days after DES administration (Fig. 2b, e).

### hAMSCs increased hormone secretion in POF mice

**Fig. 3:**
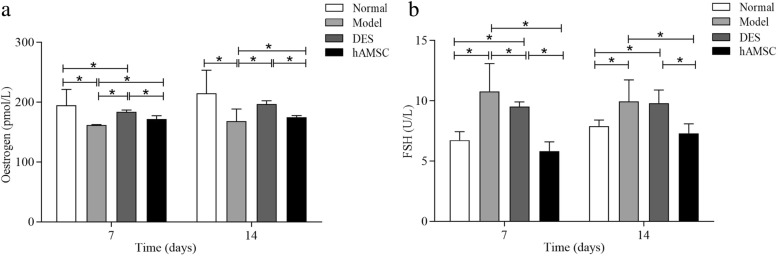
Transplantation of hAMSCs improves serum oestrogen and FSH hormone levels in POF mice. Serum levels of oestrogen (**a**) and FSH (**b**) were detected at 7 and 14 days after cells transplantation. Data are presented as the mean ± S ($\overline{x}$ ± *s*, *n* = 6), *P < 0.05

The effects of hAMSC transplantation on oestrogen and FSH hormone secretion in mice were investigated. The results showed that the serum levels of FSH in the POF model group were significantly increased compared to those of the normal group at 7 and 14 days (*P* < 0.05). Additionally, lower levels of oestrogen were observed in POF mice compared with the normal group (*P* < 0.05). The levels of serum FSH in the DES group were significantly decreased compared with the model group at 7 days but not at 14 days. The serum FSH content was higher than that of the normal group. Although the serum oestrogen content was lower than in the normal group, the oestrogen levels were increased in the DES mice compared with the model group (*P* < 0.05). Following the hAMSCs transplantation for 7 and 14 days, the serum levels of oestrogen were significantly elevated and FSH secretions were decreased in the hAMSC group compared with the model group. Compared with normal mice, the oestrogen levels remained lower in the hAMSC group. Based on the results from endocrine hormone changes, hAMSCs transplantation markedly promoted the recovery of the hypothalamus-ovary axis to regulate secretion in oxidative damage-induced POF mice (Fig. 3).

### hAMSCs increased the body weight and ovarian index of POF mice

**Fig. 4:**
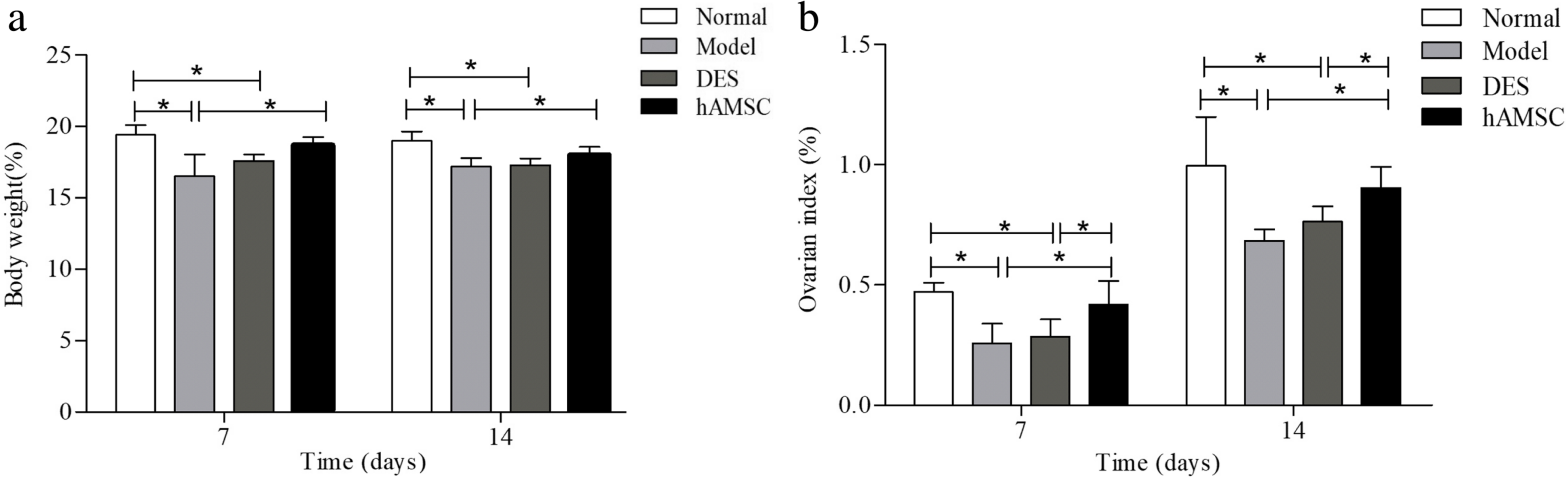
The body weight and ovarian index of POF mice were obviously increased by transplanted hAMSCs. Body weight (**a**) and ovarian index (**b**) were detected at 7 and 14 days after cells transplantation ($\overline{x}$ ± s, *n* = 6). *P < 0.05

To investigate the effects of hAMSCs transplantation on POF mice, changes in body weight and ovarian index were recorded in each group at 7 days and 14 days after hAMSCs transplantation. The body weight and ovarian index of mice in the model group were significantly decreased compared to the normal group (*P* < 0.05). In comparison, the body weight and ovarian index of mice in the DES group were significantly decreased compared with the normal group (*P* < 0.05). The body weight and ovarian index of mice were significantly improved by hAMSCs compared with the model group (Fig. 4).

### The fertility was enhanced after hAMSC transplantation

**Fig. 5:**
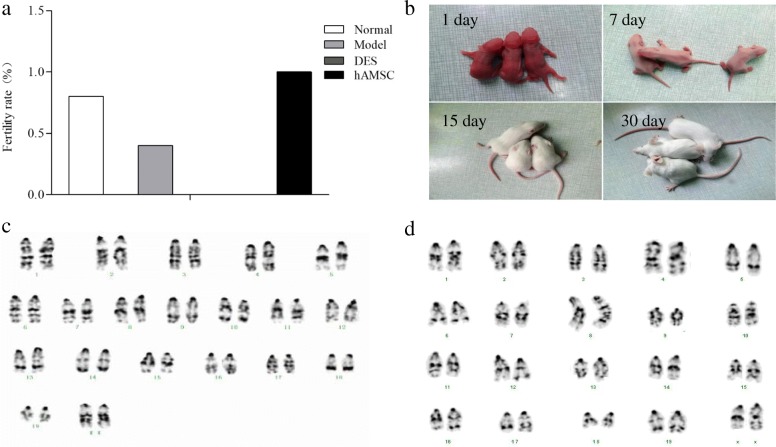
Transplanted hAMSCs improved the fertility rate in POF mice. **a** The different fertility rates are shown in each group, but the DES group showed infertility (*n* = 10). **b** The suckling mice of the hAMSC group were observed for 30 days. Karyotype analysis of the chromosomes of offspring mice in the normal group (**c**) and hAMSC group (**d**) were observed under oil immersion lenses (× 400)

On the 14th day after transplantation, mice were mated with proven fertile males for 7 days. In addition to the DES group, the other groups had different numbers of mice giving birth. The farrowing rates in the POF model group (40%) and the DES group (0) were significantly decreased compared with those in the normal group (80%). The farrowing rates of the hAMSC group (100%) were higher than those of the model and DES groups (*P* > 0.05), but there was no significant difference compared to those of the normal group. The pregnancy rates in the normal, model, DES, and hAMSC groups were 80% (8/10), 40% (4/10), 0% (0/10), and 100% (10/10), respectively. There were no significant differences in the number of foetuses per litter among the four groups (Fig. 5a).

The number born per litter in each group was close to 3 suckling mice (range 2–5). There was no obvious abnormality in the appearance of neonatal mice in each group. After 30 days, the mice in each group were similar to normal mice, and they showed normal movement, eating, and defecating. The mice grew quickly and developed and responded sensitively (Fig. 5b). After 45 days, female offspring in the hAMSC group were combined with male mice, and they were also able to reproduce as usual. Karyotype analysis of bone marrow cell chromosomes in offspring mice showed that each chromosome was clearly identifiable, and the chromosome karyotype of the offspring in the hAMSCs transplantation mice was the same as the normal mice (Fig. 5c, d), with no obvious deletions, inversions, or translocations.

### hAMSCs transplantation improved histopathological changes in ovarian tissues

**Fig. 6:**
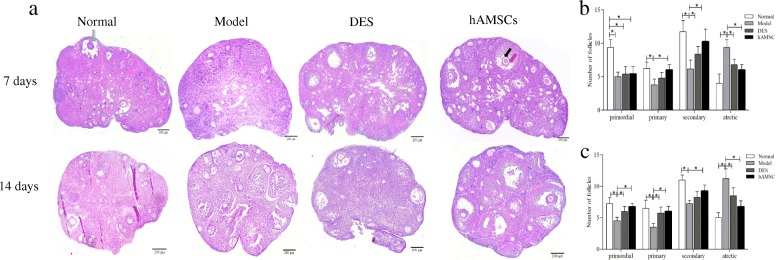
Transplantation of hAMSCs reduces ovarian injuries in POF mice. **a** The pathological changes of ovaries were evaluated by H&E staining in the four groups at 7 and 14 days after hAMSCs transplantation (× 40). Quantitation of follicle count from ovaries in mice of the four groups at 7 (**b**) and 14 (**c**) days after cell transplantation (*n* = 4). The black arrow shows granulosa cells, the red arrow shows the cumulus complex, and the green arrow shows mature follicles

Histopathological examination was conducted in all groups to evaluate the effect of hAMSCs transplantation (Fig. 6). The ovaries of normal group mice contained many healthy follicles at all stages, including primordial follicles, primary follicles, secondary follicles, and antral follicles. In contrast, the ovaries of H_2_O_2_-induced POF model mice showed atrophied ovaries, which were mostly composed of interstitial cells in a fibrous matrix, with a reduced number of follicles at each stage of development. Fewer well-developed follicles and many atresia follicles were found in the POF model mice than in the normal group. Follicles with development at all levels of the POF mice were partially restored. Specifically, a certain number of mature follicles were observed, although they did not recover to the normal level within 14 days after hAMSCs transplantation. The morphology of ovaries in the DES group was similar to that of POF model mice at 7 days after DES treatment, and there was no significant difference between the DES group and the model group at 14 days. In comparison, the ovary size of the hAMSC group was larger than that of the model group at 7 and 14 days. We also observed that follicles at all stages of development with abundant granulosa cells (Fig. 6, black arrow) were significantly increased and were different from the POF model group (Fig. 6a, b, *P* < 0.05). After 14 days, the ovary morphology of hAMSCs transplantation mice was similar to that of the normal ovaries in the normal group. There were many mature oocytes surrounded by several layers of squamous granulosa cells. The oocyte-corona-cumulus complex (Fig. 6, red arrow) and pellucid zone were all observed in mature oocytes.

### PKH26-labelled hAMSC location and distribution

**Fig. 7:**
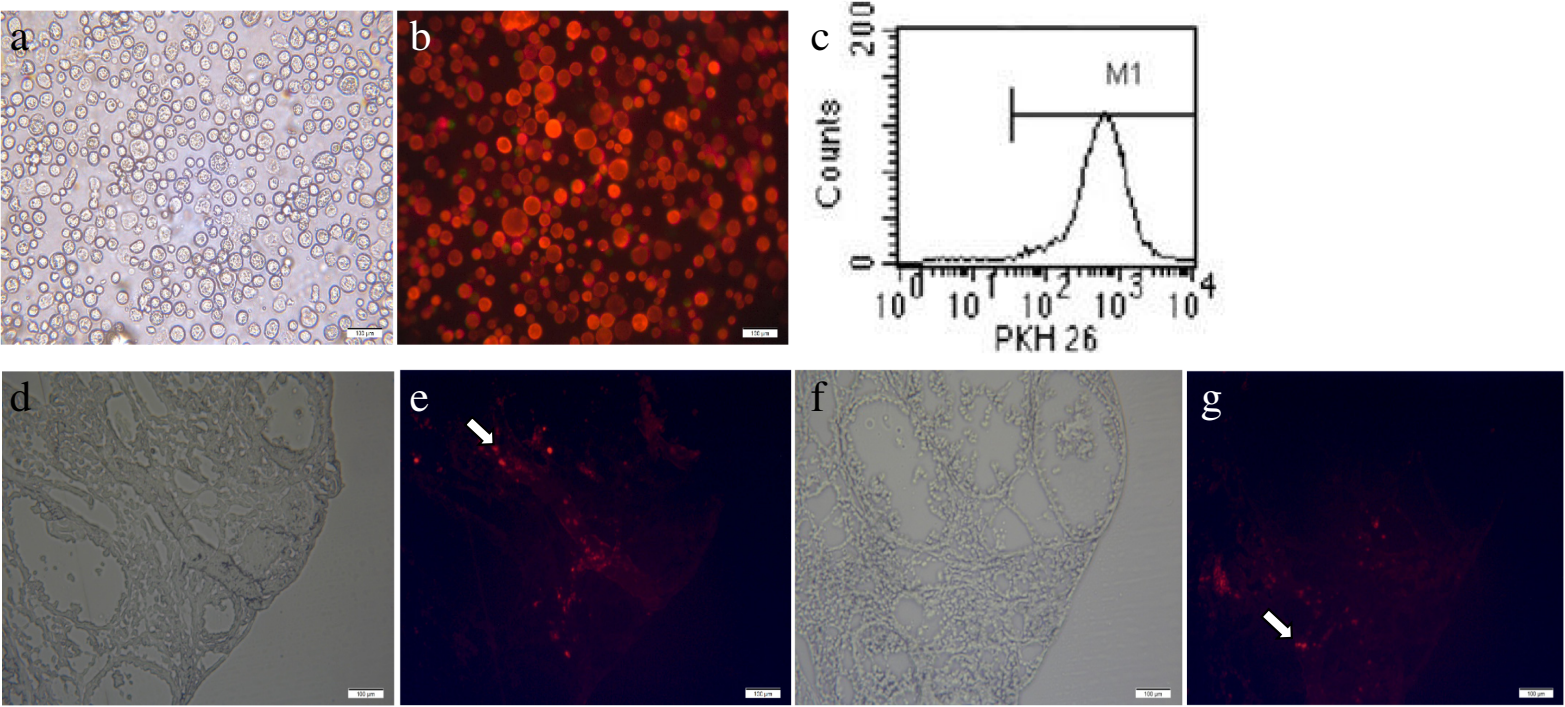
In vivo hAMSCs tracking. **a**, **b** PKH26-labelled hAMSCs showed red fluorescence (× 100). **c** The labelling rates of PKH26-labelled hAMSCs were detected by flow cytometry. Transplanted hAMSCs were observed at 7 (**d**, **e**) and 14 (**f**, **g**) days after cell transplantation in ovaries (× 100, *n* = 3). **d** and **f** show the bright field under a fluorescence microscope, and **e** and **g** show the dark field (× 100)

To trace the fate of the hAMSCs in vivo, the hAMSCs were labelled with PKH26. After labelling, the red fluorescence of PKH26 was uniformly distributed in the cell membrane. The cell contour was clear, and the membrane surface was intact under an inverted phase contrast fluorescence microscope (Fig. 7a, b). Then, the percentage of red fluorescence was detected by flow cytometry, and 99% of the hAMSCs expressed red fluorescence (Fig. 7c). Furthermore, the transplanted hAMSCs were labelled with PKH26 to trace their fate in the frozen section of ovarian tissue. The location of the PKH26-marked hAMSCs was determined by red fluorescence signals. The PKH26 of red fluorescence-labelled cells were located in the interstitium but not in follicles (Fig. 7e, g). After 14 days, the number of red fluorescent cells decreased, and the fluorescence intensity weakened (Fig. 7g). Red fluorescence was not observed in the normal, model, and DES groups.

### hAMSCs regulated the expression of five proteins

**Fig. 8:**
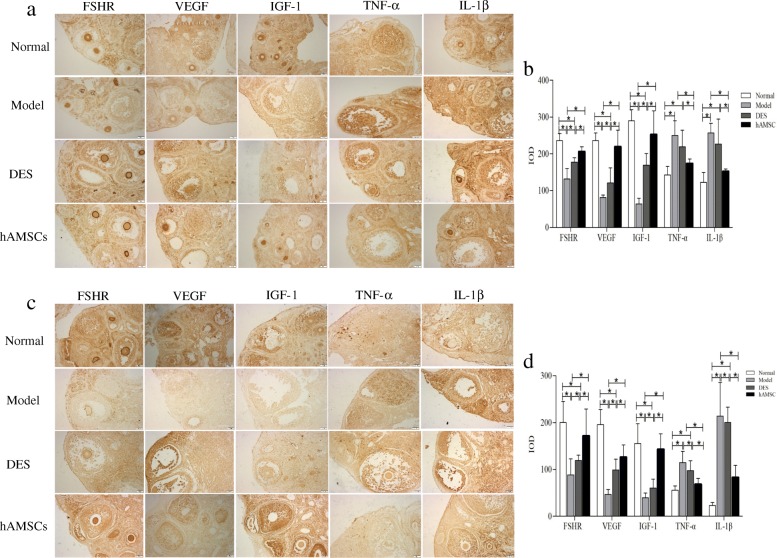
The hAMSCs improved protein expression associated with follicular development in the ovarian microenvironment of POF mice. The brown particles of positive expression were observed on FSHR, VEGF, IGF-1, TNF-α, and IL-1β proteins by immunohistochemical staining at 7 days (**a**) and 14 days (**c**) after hAMSCs transplantation. At 7 (**b**) and 14 days (**d**) after cell transplantation, the mean integral optical density value (IOD) of FSHR-, VEGF-, IGF-1-, TNF-α-, and IL-1β-positive areas was determined in ovarian tissue by Image-Pro Plus (immunohistochemical staining, × 200). Data are presented as the mean ± S ($\overline{x}$ ± *s*, *n* = 6), *P < 0.05

To gain further insight into the potential ovary reparative mechanisms of the ovarian microenvironment underlying the functional benefits observed following treatment with hAMSCs transplantation, the levels of two growth factors and the FSHR known to be associated with ovarian regeneration and two inflammatory cytokines were evaluated after immunohistochemical staining. Positive signals for expression of FSHR and IGF-1 were observed as brownish staining in the cytomembrane, whereas a positive signal for VEGF, TNF-α, and IL-1β expression was observed in the cytoplasm. In the normal ovarian tissue, FSHR was mainly expressed in ovarian stromal cells and theca cells. VEGF was mainly expressed in the stromal cells surrounding the follicle, corona radiata, granulosa cells, and theca cells. IGF-1 was expressed in the ovary stromal cells and granulosa cells (Fig. 8a, c). After treatment for 7 days, the expression levels of FSHR, VEGF, and IGF-1 in the POF model group were significantly lower, and TNF-α and IL-1β were higher than in the normal group (*P* < 0.05), while the former three factors in the hAMSC group were significantly increased compared with the model and DES groups, but the later inflammatory cytokines were tremendously reduced (*P* < 0.05, see Fig. 8b). Similar and more pronounced results were observed at 14 days after hAMSCs administration. The levels of TNF-α and IL-1β were further reduced in hAMSCs at 14 days (Fig. 8d).

### Changes in mRNA levels after hAMSCs transplantation

**Fig. 9:**
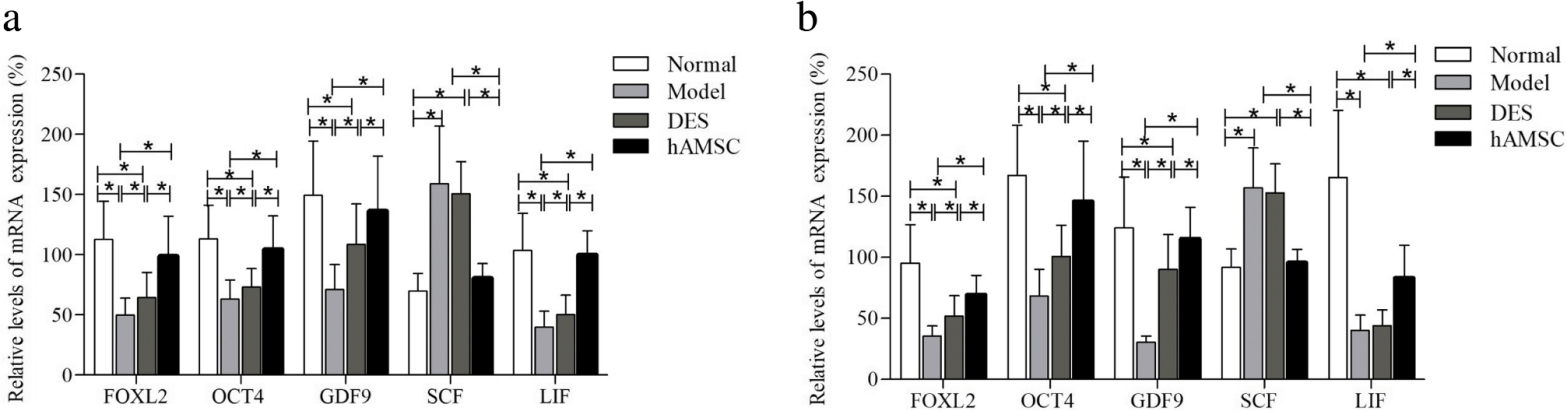
The mRNA levels of FOXL2, OCT4, GDF-9, SCF, and LIF were regulated by hAMSCs in the ovarian tissues of POF mice by real-time PCR at 7 (**a**) and 14 (**b**) days after cells transplantation. Data are presented as the mean ± S ($\overline{x}$ ± *s*, *n* = 6), *P < 0.05

The gene expression of ovarian tissue was detected to explore the mechanism of hAMSCs on restoration of ovarian function in POF mice by real-time PCR at 7 and 14 days after hAMSCs transplantation. The results showed that the mRNA levels of FOXL2, Oct4, GDF-9, and LIF were significantly downregulated in the POF model group compared with the normal group, whereas SCF was upregulated (*P* < 0.05). The mRNA expression levels of the FOXL2, Oct4, GDF-9, and LIF genes were increased in hAMSCs- and DES-treated mice compared with those in model mice, while the SCF mRNA expression was reduced. There was no significant difference in the mRNA levels of these five genes between the hAMSC and the normal groups (*P* > 0.05, Fig. 9).

## Discussion

In this study, the results indicated that hAMSCs isolated from the human amnion were multipotent MSCs with adherent growth and showed a long fusiform morphology with and without expression of CD molecules. The procedure of obtaining hAMSCs was noninvasive, safe, and out of ethical debate. hAMSCs transplantation can improve the function and structure of injured ovarian tissue in oxidatively damaged POF mice. The effects are the same as other reports about bone marrow, umbilical cord, and human placenta-derived MSCs [13, 22, 46]. Furthermore, the ovarian index, levels of FSH and oestrogen, and fertility rates in hAMSCs transplantation mice were recovered compared with model mice. The newborn mice had no obvious deformity with a normal karyotype and had normal growth, development, and fertility. PKH26 in red fluorescence-labelled hAMSCs was mainly located in the ovarian interstitium. This finding suggests that hAMSCs did not differentiate into follicular cells or granulosa cells. The mechanisms of hAMSCs may be related to facilitating follicular development, granulosa cell proliferation, and secretion function by improving the local microenvironment of the ovary.

H_2_O_2_ is an endogenous reactive oxygen species, and strong oxidants can cause DNA damage, apoptosis, tissue injury, and inflammation; accelerate ageing; and increase the risk of cancer. H_2_O_2_ is associated with a variety of tissue and organ degenerative diseases [47–49]. Thus, we established the POF model in mice using 10% H_2_O_2_ treatment to cause ovarian injury. After 7 days, the mice with a side ovary that was burnt with 10% H_2_O_2_ lost a significant amount of body weight and showed a decreased ovary index. We also found that the oestrus cycle was clearly disorganized through the evaluation of the oestrus cycle. Histological analyses indicated that the numbers of follicles in various stages of development were clearly reduced, the cortex of the ovarian tissue was thin, and the interstitial part was obviously loose and fibrosis. We demonstrated that ovarian function was significantly damaged, and the POF model was successfully established through this approach. In addition, the study showed that serum FSH was significantly increased while serum oestrogen was obviously decreased compared with the normal group. These data are consistent with the POF disorder.

In our study, hAMSCs isolated from human amnions had the common characteristics of MSCs. The cell-surface markers of more than 95% of the hAMSCs were positive for CD44, CD90, CD105, and CD73 and negative for CD45, CD34, CD11b, CD19, and HLA-DR. In addition, hAMSCs showed fibroblast-shaped morphology and adherence to plastic. These findings met the criteria for MSCs identification by The Association of International Cell Therapy. PKH26-labelled hAMSCs have better growth for tracers in the body. The labelling rate was 99%. These hAMSCs were transplanted into POF mice. Following hAMSCs transplantation for 7 days, the hAMSC group showed major improvements in the function and structure of ovarian tissue compared to the model group, which included dramatically increased body weight and ovary index, higher recovery rate of regular oestrus cycle, increased healthy follicle numbers, significant elevation of oestrogen production, and reduced release of FSH. At 14 days after hAMSCs transplantation, ovarian function recovery was more significant and almost normal. The pregnancy rate in the hAMSC group was 100%. There was no obvious abnormality in newborn mice, which had normal chromosome karyotypes and no obvious abnormalities. The offspring were also normally fertile. The efficacy of hAMSCs was superior to DES. DES did not improve normal follicular development and fertility because the DES replacement therapy could not obviously promote the repair of ovarian damage. Therefore, DES only increased the level of oestrogen in serum and decreased the level of FSH in serum at 7 days, but the level of FSH was not obviously decreased at 14 days. FSH could stimulate follicle development and granulosa cells growth. Granulosa cells produce oestrogen. With the increased level of oestrogen within limits, the levels of serum FSH should be decreased by negative feedback regulation of the hypothalamic-pituitary-gonadal axis, thus reducing follicle excessive growth and avoiding their depletion. The results may be involved in the established inadequacy and unstable negative feedback of the hypothalamic-pituitary-gonadal axis with DES treatment. These data strongly indicated that hAMSCs successfully recovered ovarian function and negative feedback regulation of the hypothalamic-pituitary-gonadal axis in oxidative damage-induced POF mice.

Follicular growth requires systemic regulation by hormones and intraovarian regulation by cytokines, growth factors, and intracellular proteins. To clarify the mechanism of hAMSCs in repairing the damaged ovarian function in POF mice, we observed the distribution of hAMSCs in vivo. PKH26-labelled hAMSCs were transplanted into injured ovaries. The transplanted hAMSCs were mainly located in the interstitial tissues of the ovary after 7 days, which was similar to previous reports [11, 19, 20]. The results indicated that hAMSCs may restore ovarian function of H_2_O_2_-induced POF through paracrine effects rather than through differentiation into granulosa cells or oocytes. hAMSCs were shown to secrete various cytokines, such as TGF-β, HGF, IGF-1, VEGF, EGF, HB-EGF, and bFGF [29, 33]. In our study, we focused on IGF-1 and VEGF, which are likely to play important roles in restoring ovarian function in POF animals via stem cells. The results showed that the expression of VEGF and IGF-1 proteins was upregulated in the ovary tissue after hAMSCs transplantation. We further showed that hAMSCs could improve the local microenvironment and inhibit granulosa cell apoptosis induced by H_2_O_2_ in ovarian tissue. Thus, we speculate that the repaired ovarian tissue damage and improved ovarian function are likely to be partially mediated by the growth factors produced by hAMSCs through the paracrine pathway.

In mammalian ovaries, the FSH-specific transmembrane receptor FSHR is mainly located in granulosa cells as a common marker gene and is an important modulator of granulosa cell function. FSHR is an antiapoptotic factor in granulosa cells. Activation of FSHR signalling strengthens granulosa cell proliferation and function [50]. The results showed that expression of FSHR was upregulated in the ovary tissue after hAMSCs transplantation, especially on the granulosa cells. Thus, we speculate that hAMSCs increases the expression of FSHR in the damaged ovary, thereby promoting the proliferation of granulosa cells and inhibiting follicular atresia to restore ovarian function.

The mammalian ovary has a very complex machinery, in which many factors play an important role in the maturation and growth of oocytes during folliculogenesis. FOXL2 is expressed throughout ovarian development. However, FOXL2 function in the ovary first becomes essential during initial primordial follicle recruitment after birth. The FOXL2 gene is essential for granulosa cell differentiation and ovarian function maintenance [51]. FOXL2 is expressed in the less differentiated granulosa cells of small and medium follicles. Therefore, it likely also plays a role in granulosa cell differentiation and follicle development and maintenance. Some studies indicate that FOXL2 functions as a suppressor of ovarian follicle progression in small and medium follicles by preventing premature differentiation and/or proliferation of granulosa cells, thus preventing premature depletion. Furthermore, FOXL2, a key modulator of steroidogenesis, is involved in oestrogen and progesterone production [52, 53]. Oestrogen itself plays important roles in regulating the development and function of granulosa cells as well as the development of follicles [54]. Oct4 is also known as OTF3 and POU5F1. This protein is a POU-domain, octamer-binding transcription factor expressed in both mouse and human embryonic stem cells and primordial germ cells [55]. A potential role for Oct4 is the recruitment of oocytes for their maturation. Overexpression of Oct4 in porcine ovarian stem/stromal cells enhances the differentiation of oocyte-like cells in vitro and ovarian follicular formation in vivo [56]. Growth differentiation factor-9 (GDF-9) genes are relevant members of the TGF-β superfamily that encode proteins secreted by the oocytes into the ovarian follicles [57]. GDF-9 is necessary to optimize the oocyte microenvironment, ovarian follicle growth and atresia, ovulation, fertilization, and normal reproduction [58]. GDF-9 plays an important role in oocyte development. It contributes to promoting the proliferation and metabolism of granulosa cells and stimulates the expression of kit ligand on granulosa cells. The kit ligand acts on its receptor on the oocyte and modulates oocyte development. Previous studies have found that the expression of GDF-9 mRNA in cumulus granulosa cells was positively correlated with oocyte maturation, normal fertilization rate, cleavage rate, embryo quality, and clinical pregnancy outcome. Thus, the levels of GDF-9 mRNA in cumulus granulosa cells may be considered new biomarkers for predicting oocyte developmental potential [59]. LIF is expressed in the ovary and controls follicular growth. Studies have shown that LIF coordinates the sequence of follicular growth and ovulation and plays a role in the local regulation of follicular growth in the ovary [60]. After transplanted hAMSCs dramatically increased the levels of FOXL2, Oct4, GDF-9, and LIF mRNA, their coded proteins obviously promoted the recruitment of primitive follicles and the orderly development of primary follicles into secondary follicles and mature follicles. In addition, hAMSCs secrete GDF-9 and LIF and other somatomedin. They may also provide good endometrium conditions for implantation, cleavage, and embryo development of fertilized eggs. Then, the fertility of POF was restored. This is another obvious advantage of hAMSCs over DES.

SCF exerts its biological effects by binding to the tyrosine kinase receptor c-Kit, which is located on the cell surface. Previous studies have suggested that c-Kit and SCF play important roles during migration of primordial germ cells into the gonadal anlage and during oogenesis and folliculogenesis in the human foetal ovary [61]. SCF is an important regulator of ovarian development in embryos and adults. SCF promotes follicular growth by stimulating the function of thecal-interstitial cells through the Erk1/2 pathway [62]. Our results showed that the level of SCF mRNA in ovarian tissue was significantly decreased to the level of normal mice in the hAMSC group, but SCF was increased in the DES group, similar to the model group. This may be consistent with the high expression in the early and middle stages of follicle formation, while the level is reduced after follicle development to maturation. It also illustrates the difference between hAMSCs and DES at the molecular level in that the former not only can promote follicular development but also promote secondary follicle development into mature oocytes by restoring the function of granulosa cells to form dominant follicles and ovulation. The coordinating changes in the expression of these genes also explained the histopathological difference between the POF mice treated with hAMSCs transplantation and DES. Therefore, the sustained low expression of FOXL2, Oct4, GDF-9, and LIF in the ovaries of POF mice significantly inhibited the further development of primary follicles, while the persistently high expression of SCF may be an important factor affecting the development and maturation of secondary follicles. The fine regulation of the above important proteins and mRNAs in the ovarian microenvironment may be an important target for further investigation. The advantages of hAMSCs are also reflected to some extent in the precise and more comprehensive microenvironment regulation.

## Conclusions

This study revealed that hAMSCs significantly improved ovarian function and histopathological structure and promoted follicle development and maturation in ovarian injury of H_2_O_2_-induced POF mice. The therapeutic effect of hAMSCs on POF was better than that of DES. The results suggest a potential mechanism for hAMSCs treatment involving the amelioration of micro-circumstances by paracrine effects of planting hAMSCs. One of the most important changes was that the expression levels of the FSHR, VEGF, IGF-1, TNF-α, and IL-1β proteins and FOXL2, Oct4, GDF-9, LIF, and SCF mRNA were precisely regulated in ovarian tissues by hAMSCs. The hAMSCs and the secreta transplantation also represent an alternative and extraordinarily promising strategy for POF therapy.

## Data Availability

Data and materials used and/or analysed during the current study are available from the corresponding author on reasonable request.

## Abbreviations

AMH: Anti-Müllerian hormone
DES: Diethylstilbestrol
FOXL2: Forkhead box L2 gene
FSH: Follicle-stimulating hormone
FSHR: Follicle-stimulating hormone receptor
GDF-9: Growth differentiation factor-9
H_2_O_2_: Hydrogen peroxide
hAMSCs: Human amnion-derived mesenchymal stem cells
IGF-1: Insulin-like growth factor-1
IL-1β: Interleukin-1β
IOD: Integral optical density value
LIF: Leukaemia inhibitory factor
MSCs: Mesenchymal stem cells
Oct4: Octamer combination transcription factors
POF: Premature ovarian failure
SCF: Stem cell factor
TNF-α: Tumour necrosis factor
VEGF: Vascular endothelial growth factor
